# Conglomerate, Racemate, and Achiral Crystals of Polymetallic Europium(III) Compounds of Bis- or Tris-β-diketonate Ligands and Circularly Polarized Luminescence Study

**DOI:** 10.1021/acsomega.2c07310

**Published:** 2023-01-31

**Authors:** Marine Louis, Yan Bing Tan, Pablo Reine, Shohei Katao, Yoshiko Nishikawa, Fumio Asanoma, Tsuyoshi Kawai

**Affiliations:** Graduate School of Science and Technology, Division of Materials Science, Nara Institute of Science and Technology, NAIST, 8916-5 Takayama, Ikoma, Nara 630-0192, Japan

## Abstract

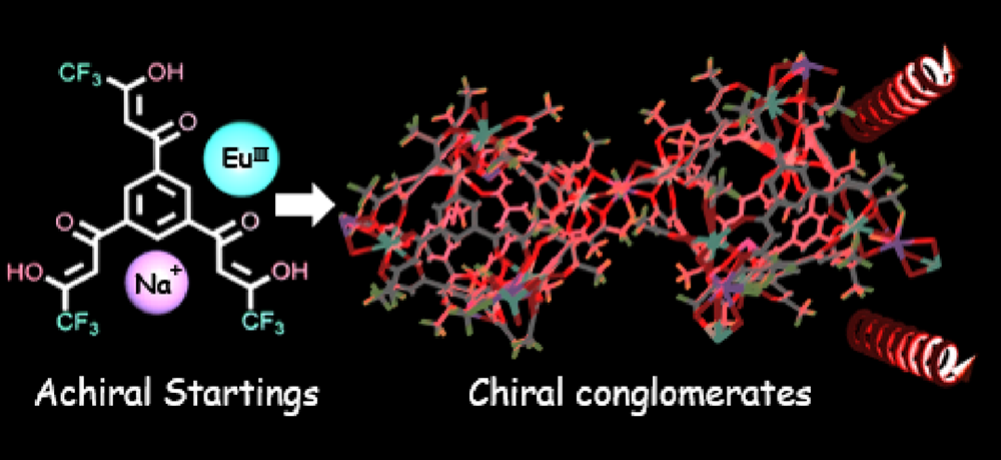

This work reports (a) conglomerate and racemic crystal structures of [(Δ,Δ,Δ,Δ,Δ,Δ)- or/and (Λ,Λ,Λ,Λ,Λ,Λ)-Eu^III^_6_(TTP)_8_(OH_2_)_6_Na_4_]_*n*_ coordination polymers, (b) racemic crystal structures of (Δ,Δ,Δ,Δ)-/(Λ,Λ,Λ,Λ)-Eu^III^_4_(TTP)_4_(bipy)_4_(MEK)_2_(OH_2_)_2_ tetrahedral clusters, and (c) the achiral crystal structure of the [Eu^III^_2_(BTP)_4_(OH_2_)_2_Na_2_]_*n*_ coordination polymer (where BTP = dianionic bis-β-diketonate, TTP = trianionic tris-*β*-diketonate, and bipy = 2,2′-bipyridine). The screw coordination arrangement of the TTP ligand has led to the formation of homoconfigurational racemic Eu^III^ products. The conglomerate crystallization of [Eu^III^_6_(TTP)_8_(OH_2_)_6_Na_4_]_*n*_ appears to be caused by the presence of the sodium, Na^+^ counterions, and interactions between oxygen atoms and the trifluoromethyl unit of the TTP ligand and Na^+^ ions. All the Eu^III^ compounds exhibit characteristic red luminescence (^5^D_0_ → ^7^F_J_, *J* = 0–4) in solution or in the solid crystalline state. Circularly polarized luminescence (CPL) was observed in the chiral Eu^III^_6_(TTP)_8_(OH_2_)_6_Na_4_]_*n*_ species, displaying a |*g*_lum_| value in the range of 0.15 to 0.68 at the ^5^D_0_ → ^7^F_1_ emission band. Subtle changes of the [Eu^III^_6_(TTP)_8_(OH_2_)_6_Na_4_]_*n*_ structure which may be due to selection of twinned crystals or crystals that do not correspond to a perfect spontaneous resolution, are considered to be responsible for the variation in the observed CPL values.

## Introduction

Induction and control of chirality are the central topics of organic, polymer, and inorganic chemistry. Chiral coordination complexes have been extensively developed and acknowledged for their key role in stereoselective synthesis, which has been rewarded the 2001 Chemistry Nobel Prize given to Noyori, Knowles, and Sharpless.^1−5^ Chiral polymers are widely used in high-performance liquid chromatography (HPLC) for the separation of chiral substances.^6−8^ Recently, chiral substances have also been in the spotlight for their promising properties as chiral light emitters. Circularly polarized luminescence (CPL) has attracted considerable attention for applications in optoelectronics, OLEDS, security tags, or luminescent probes.^9−16^ Chiral coordination substances based on d-group and f-group elements, such as Pt(III), Ru(II), Au(I), Eu(III), Tb(III), and others have been widely investigated.^17−33^ Recently, metal clusters have also been reported for their CPL capability.^34−36^ Several approaches are considered to systematically induce chirality in the coordination complexes: intrinsic chirality of the metal clusters,^37,38^ the use of chiral ligands,^23,39,40^ or the generation of an asymmetric arrangement around the metal center using an achiral ligand. The synthesis of many CPL-active enantiopure complexes relies on the second approach. The latter method, less predictable, often leads to a racemic mixture, which can be quite arduous to separate, requiring specialized techniques such as chiral HPLC or chiral ion-pairing. Particularly for species such as lanthanide complexes, which possess inherent kinetic lability and generally have a rather fluxional coordination sphere,^41^ the spontaneous resolution of enantiomers, also known as conglomerates, remains to be the most challenging but promising route to obtain CPL materials. In the last decade, spontaneous resolution of conglomerate lanthanide(III) (Ln^III^) coordination complexes and coordination polymers has been reported based on multi-dentate ligands.^42−49^ Intermolecular interactions observed in the Ln^III^ crystal structures predominantly govern the conglomerate crystallization process. Despite several examples of solid-state circular dichroism, CD,^42,44−47^ the CPL of conglomerate crystals has only been reported by Zhu et al. in a chiral molecular organic framework (CMOF).^49^ Due to significant advantages of low costs, high efficiency, and easy scale-up, the conglomerate approach of CPL lanthanide complexes is desired to adapt to the solution-phase processability.

**Figure 1:**
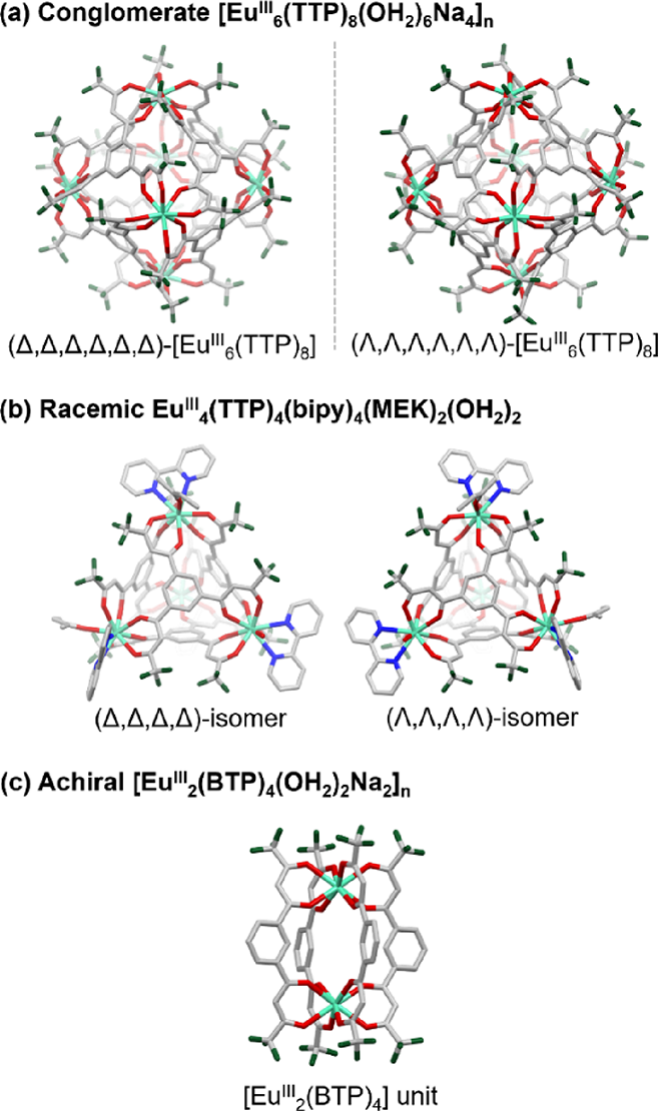
(a) Conglomerate crystal structures of [(Δ,Δ,Δ,Δ,Δ,Δ)- or (Λ,Λ,Λ,Λ,Λ,Λ)-Eu^III^_6_(TTP)_8_(OH_2_)_6_Na_4_]_*n*_. Only a [Eu^III^_6_(TTP)_8_] unit is shown. (b) Racemic crystal structures of (Δ,Δ,Δ,Δ)-/(Λ,Λ,Λ,Λ)-Eu^III^_4_(TTP)_4_(bipy)_4_(MEK)_2_(OH_2_)_2_. (c) Crystal structure of [Eu^III^_2_(BTP)_4_(OH_2_)_2_Na_2_]_*n*_. Only a [Eu^III^_2_(BTP)_4_] unit is shown. Solvents, hydrogen atoms, and/or Na^+^ counterions have been omitted for clarity.

We here report chiral Eu(III) coordination polymers based on conglomerate crystallization and their CPL activity in solid powders and the solution phase. New Eu^III^ coordination polymers are composed of a tris-β-diketonate ligand (1,3,5-tris(3-trifluoromethyl-3-oxopropanoyl) benzene, H_3_TTP). [(Δ,Δ,Δ,Δ,Δ,Δ)- and (Λ,Λ,Λ,Λ,Λ,Λ)-Eu^III^_6_(TTP)_8_(OH_2_)_6_Na_4_]_*n*_ (Figure 1a and Figure S1) display CPL profiles in the crystalline state and similarly in the solution phase, which indicate reliability of the solid-state CPL data and good configurational stability in the solution phase. The preferential self-sorting crystallization process of [Eu^III^_6_(TTP)_8_(OH_2_)_6_Na_4_]_*n*_ is believed to be associated with CF–Na^+^ interactions between oxygen atoms and the trifluoromethyl unit of the TTP ligand and Na^+^ ions. We also observed a racemic crystal for the same stoichiometric composition. Different stoichiometry ratios of Eu^III^ and TPP derive a racemate tetrahedral tetranuclear Eu^III^ cluster with a bypyridine (bipy) ligand and methyl ethyl ketone (MEK), namely, (Δ,Δ,Δ,Δ)-/(Λ,Λ,Λ,Λ)-Eu^III^_4_(TTP)_4_(bipy)_4_(MEK)_2_(OH_2_)_2_ (Figure 1b). An achiral crystal of the Eu^III^ coordination polymer of the bis-β-diketonate (1,3-bis(3-trifluoromethyl-3-oxopropanoyl) benzene, H_2_BTP) ligand,^40^ that is, [Eu^III^_2_(BTP)_4_(OH_2_)_2_Na_2_]_*n*_, is also prepared as a reference to study the coordination mode of Na^+^ counterions to the main dinuclear Eu^III^ frame unit (Figure 1c).

## Results and Discussion

### Crystal Structure Descriptions

**Figure 2:**
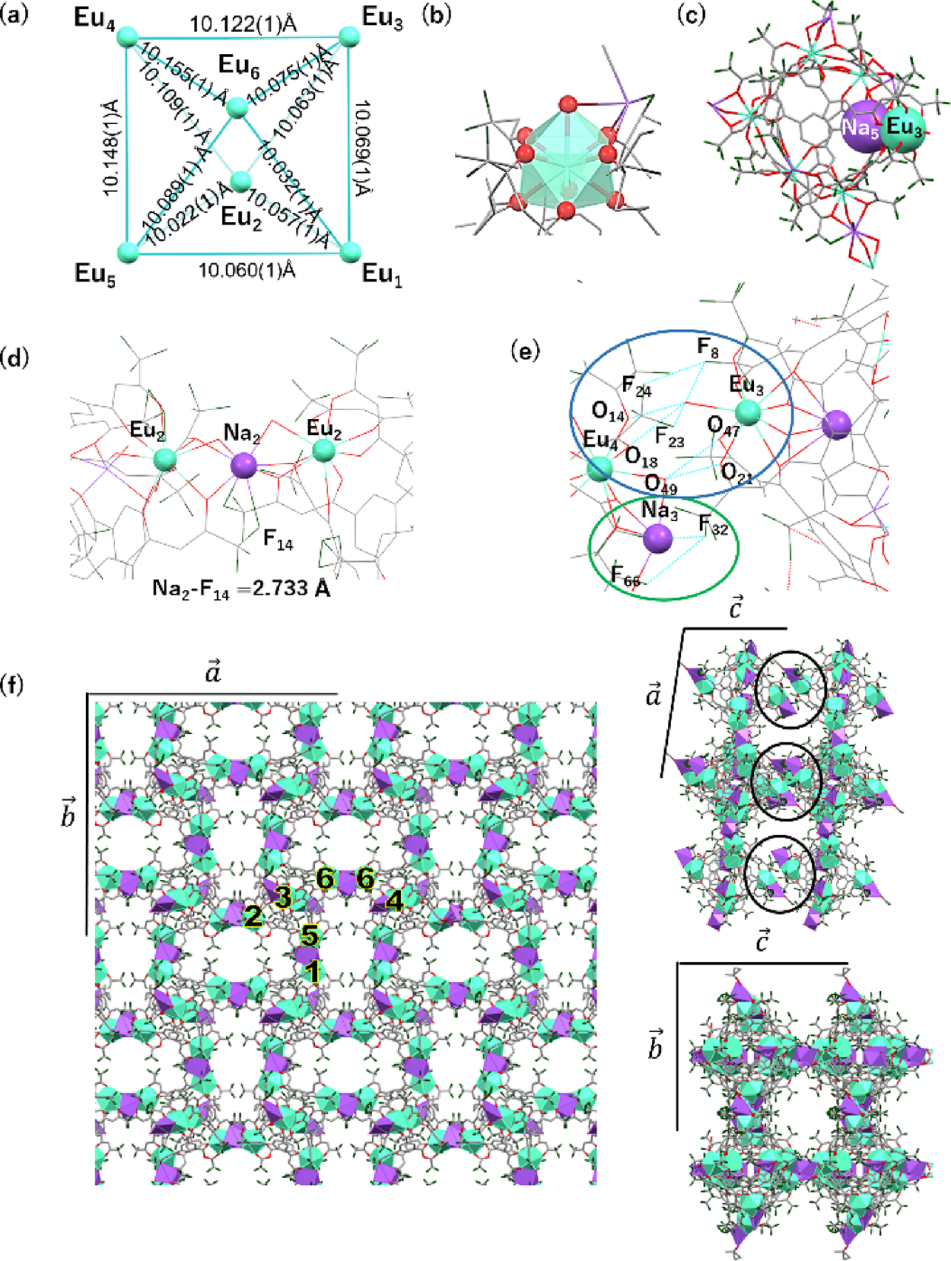
(a) Hexanuclear cage unit of (Δ,Δ,Δ,Δ,Δ,Δ)-[Eu^III^_6_(TTP)_8_] with Eu–Eu distances. (b) Eu^III^ polyhedral of [(Δ,Δ,Δ,Δ,Δ,Δ)-Eu^III^_6_(TTP)_8_(OH_2_)_6_Na_4_]_*n*_. (c) Sodium ion Na_5_ located inside the cage. (d) Eu–Na–Eu connection in [(Δ,Δ,Δ,Δ,Δ,Δ)-Eu^III^_6_(TTP)_8_(OH_2_)_6_Na_4_]_*n*_. (e) View along the *b* axis; inter-nanosheet interactions. Short contacts are indicated in light-blue dotted lines. Inter-sheet inter-ligand interactions are encircled in blue, and CF_3_(TTP)-Na_3_ interacted units between the 2D nanosheets are encircled in green. (f) Crystallographic packing of the [(Δ,Δ,Δ,Δ,Δ,Δ)-Eu^III^_6_(TTP)_8_(OH_2_)_6_Na_4_]_*n*_ coordination polymer. (Left) Packing structures viewed along the *c* axis where a coordination polymer forms the 2D sheet of a square grid. (Top right) Packing structures viewed along the *b* axis with interacting inter-sheet units encircled in black. (Bottom right) Packing structures viewed along the *a* axis. Light-green and purple polyhedrons indicate Eu^III^ and Na^+^ ions, respectively. The numbers in the light-green polyhedral denote the labeling of Eu^III^ ions. Solvents and hydrogen atoms are omitted for clarity.

The [Eu^III^_6_(TTP)_8_(OH_2_)_6_Na_4_]_*n*_ was obtained from the reaction between Eu^III^ chloride hexahydrate and H_3_(TTP) in a 3:4 stoichiometry ratio in the presence of sodium hydroxide. Single crystals were successfully grown from slow diffusion of diethyl ether into a solution containing the sample in acetone. Several attempts (12 times) in crystallization resulted mostly in conglomerate crystal structures of homoconfigurational (Δ,Δ,Δ,Δ,Δ,Δ)- or (Λ,Λ,Λ,Λ,Λ,Λ)-[Eu^III^_6_(TTP)_8_] coordination polymers (Figure 1a, Figure S1, and Table 1 for three sets of crystallographic data). The chiral conglomerate has a Flack parameter in the range of 0.220(4) to 0.480(4), confirming highly enantiopure crystal structures. A (Δ,Δ,Δ,Δ,Δ,Δ)-[Eu^III^_6_(TTP)_8_] monomer unit exhibits a distorted capped square antiprism (CSAP) geometry in their coordination sphere where nine vertices are saturated with eight β-diketonate oxygen atoms and an oxygen atom of a coordinating water molecule (Figure 2a,b) with an average Eu–Eu distance of 10.092 Å, which seems short enough for an Eu–Eu exciton interaction.^50,51^ The Eu–O(β-diketonate oxygen) and Eu–O(OH_2_) distances are in the range of 2.378(7)–2.493(7) Å and 2.450(6)–2.548(7) Å, respectively. The total charges of all Eu^III^ and shared Na^+^ ions in a [Eu^III^_6_(TTP)_8_] unit are −6 and 5, respectively. Other sodium ions bridge two Eu cites of adjunctive [Eu^III^_6_(TTP)_8_] cages through interactions with oxygen and CF_3_ units of the TTP ligand and water molecules (Figure 2d, Table S1) in a compressed octahedral geometry, assembling [Eu^III^_6_(TTP)_8_] units into a 2D coordination polymer, which spreads on the *a*–*b* plane (Figure 2f). A 2D polymeric structure was also reported by Yang et al.^52^ The Eu^III^–Na^+^ distances are in the range of 3.405(6)–3.568(4) Å (Table S1). Eu_1_, Eu_2_, Eu_5_, and Eu_6_ ions of a [Eu^III^_6_(TTP)_8_] unit are connected to Eu_5_, Eu_2_, Eu_1_, and Eu_6_ ions of another unit by Na^+^ ions, respectively. Conversely, Eu_3_ and Eu_4_ ions are not connected to other Eu ions. The remaining Na^+^ ions are observed between the 2D nanosheet, which are tethered to a [Eu^III^_6_(TTP)_8_] unit by a CF–Na^+^ interaction (Figure 2e).

**Table 1:** Crystallographic Parameters and Refinement Details for [(Δ,Δ,Δ,Δ,Δ,Δ)-Eu^III^_6_(TTP)_8_(OH_2_)_6_Na_4_]_*n*_ (CCDC 2045299 and CCDC 2039468) and [(Λ,Λ,Λ,Λ,Λ,Λ)-Eu^III^_6_(TTP)_8_(OH_2_)_6_Na_4_]_*n*_ (CCDC 2039467)

	[Eu^III^_6_ (TTP)_8_(OH_2_)_6_Na_4_]_*n*_		
CCDC no.	2045299	2039468	2039467
(Δ,Δ,Δ,Δ,Δ,Δ) or (Λ,Λ,Λ,Λ,Λ,Λ)	(Δ,Δ,Δ,Δ,Δ,Δ)	(Δ,Δ,Δ,Δ,Δ,Δ)	(Λ,Λ,Λ,Λ,Λ,Λ)
formula sum	[C_144_H_60_Eu_6_F_72_Na_4_O_54_]		
formula weight	5025.64		
crystal system	monoclinic		
space group	C2		
*a* (Å)	37.8779(7)	37.7425(8)	37.7895(15)
*b* (Å)	37.8173(7)	37.7166(7)	37.7376(16)
*c* (Å)	19.7975(4)	19.8129(4)	19.7925(8)
α (deg)	90.000	90.000	90.000
β (deg)	97.945(7)	97.955(7)	97.917(7)
γ (deg)	90.000	90.000	90.000
*V* (Å^3^)	28086.5(10)	27932.6(11)	27,957(2)
*T* (K)	123.15	123.15	123.15
*Z*	2	2	2
ρ calcd (g cm^–3^)	1.216	1.222	1.195
R1 [*I* > 2σ**(***I*)]	0.0506	0.0623	0.0734
wR2 [*I* > 2σ**(***I*)]	0.1141	0.1546	0.1715
Flack parameter	0.346(4)	0.220(4)	0.480(4)

**Figure 3:**
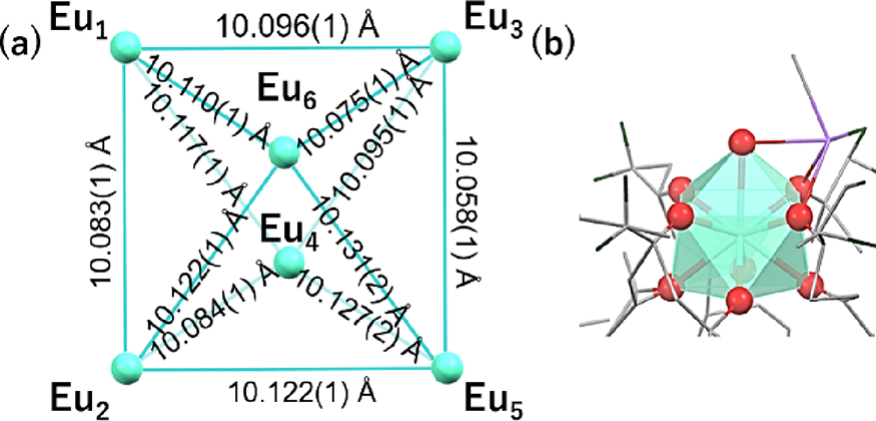
Crystal structure of a racemic crystal of [Eu^III^_6_(TTP)_8_(OH_2_)_6_Na_4_]_*n*_. (a) Hexanuclear cage unit with Eu–Eu distances. (b) Eu^III^ polyhedral of [Eu^III^_6_(TTP)_8_]. (See also Figures S1 and S2.)

We also obtained achiral [Eu^III^_6_(TTP)_8_(OH_2_)_6_Na_4_]_*n*_ crystals where (Λ,Λ,Λ,Λ,Λ,Λ)- and (Δ,Δ,Δ,Δ,Δ,Δ)-[Eu^III^_6_(TTP)_8_] hexacore cages form a 1:1 racemic crystal. Unfortunately, we could not obtain a fully refined analysis because of crystallographic disorder in this racemate. However, this still gives us precious information on the real structure of such crystals as shown in Figure 3 (see also Figure S2a,b and Table S2). The trigonal antiprism geometry is preserved with average Eu–Eu distances of 10.104 Å (Figure 3a,b). A partial 1D polymer framework is clearly observed with bridging Na^+^ ions between Eu_2_ atoms of (Λ,Λ,Λ,Λ,Λ,Λ)-[Eu^III^_6_(TTP)_8_] units with Eu_3_ atoms of (Δ,Δ,Δ,Δ,Δ,Δ)-[Eu^III^_6_(TTP)_8_] units through interactions with the oxygen atom and CF_3_ group of the TTP ligand in a similar fashion compared with the conglomerate structure (Figure S2b,c). The emergence of polymorphic architectures reminds the authors the importance of kinetic control of crystal nucleation and growth. Both types of [Eu^III^_6_(TTP)_8_] units seem to co-exist in the solution phase; between which, interconversion is slower than the nucleation and growth.^53^

**Figure 4:**
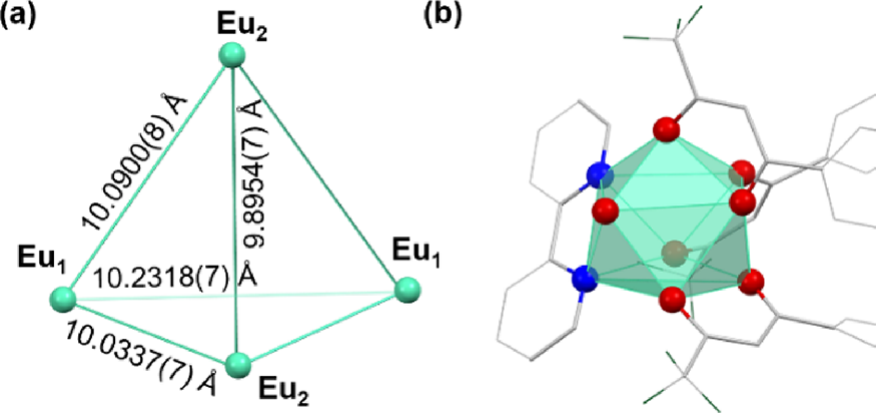
(a) Tetrahedron tetranuclear unit of (Λ,Λ,Λ,Λ)-Eu^III^_4_(TTP)_4_(bipy)_4_(MEK)_2_(OH_2_)_2_ with Eu–Eu distances. (b) Eu^III^ polyhedral of (Λ,Λ,Λ,Λ)-Eu^III^_4_(TTP)_4_(bipy)_4_(MEK)_2_(OH_2_)_2_.

We further conducted reactions between the Eu^III^ chloride hexahydrate and H_3_(TTP) in a 1:1 stoichiometry ratio in basic conditions at room temperature to prepare the precursor powders of Eu^III^_4_(TTP)_4_(sol)_*n*_ (sol = OH_2_ or CH_3_OH), which were not characterized. We tried to grow crystals from dimethoxylethane (DME) and a hexane solvent system and obtained a 1:1 racemic crystal of (Δ,Δ,Δ,Δ)- and (Λ,Λ,Λ,Λ)-Eu_4_(TTP)_4_(DME)_4_(sol)_4_. Due to high disorder of the DME molecules, the crystal structures are poorly resolved and are shown in Figure S3. Despite the insufficient structural characterization of the precursor, we could reliably use it for the sequential reaction with 2,2′-bipyridine (bipy) in a 1:4 stoichiometry ratio to form other 1:1 racemic crystals of (Δ,Δ,Δ,Δ)- and (Λ,Λ,Λ,Λ)-Eu^III^_4_(TTP)_4_(bipy)_4_(MEK)_*n*_, which crystallized in a tetragonal system with the space group *P*4̅*c*2 (Figure 1b, Figure 4, and Table S4). The racemic crystal structures exhibit a Flack parameter of 0.008(2). They possess a nearly *T*-symmetrical tetrahedral architecture where four nona-coordinated Eu^III^ ions occupy the apexes of the tetrahedron and four trianionic TTP ligands make up the four triangular faces (Figure 4a). The average Eu–Eu distance is 10.062 Å. Each nona-coordinated Eu^III^ ion exhibits a distorted CSAP geometry where nine vertices are occupied with two nitrogen atoms of the bipyridine ligand, six oxygen atoms of three β-diketonate moieties of THP ligands, and an oxygen atom of the coordinating solvent molecule (MEK) (Figure 4b). CF–F interactions between THP ligands (Figure S6) appear to stabilize the final *T* symmetrical complexes in the solid-state and solution (Figures S4d and S5d).

**Figure 5:**
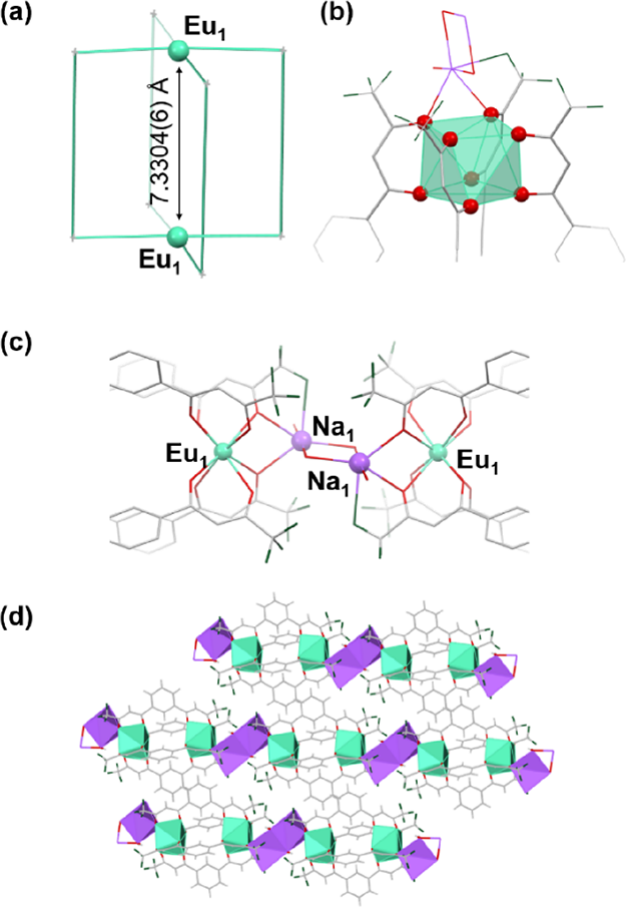
(a) Schematic dinuclear [Eu^III^_2_(BTP)_4_] unit with the Eu–Eu distance. (b) Eu^III^ polyhedral of [Eu^III^_2_(BTP)_4_(OH_2_)_2_Na_2_]_*n*_. (c) Eu–Na–Na–Eu connection in [Eu^III^_2_(BTP)_4_(OH_2_)_2_Na_2_]_n_. (d) Crystallographic packing of the [Eu^III^_2_(BTP)_4_(OH_2_)_2_Na_2_]_*n*_ coordination polymer. Solvents have been omitted for clarity. Light-green and purple polyhedrons indicate Eu^III^ and Na^+^ ions, respectively.

Another reference substance [Eu^III^_2_(BTP)_4_(OH_2_)_2_Na_2_]_*n*_ was produced from the reaction between Eu^III^ chloride hexahydrate and H_2_(BTP) in a 1:2 stoichiometry ratio in basic conditions, which was successfully crystallized in a triclinic system with the space group *P*1̅ from a solvent diffusion technique (acetone and chloroform; Figure 1c, Figure 5, and Table S4). A Eu^III^_2_(BTP)_4_(OH_2_)_2_Na_2_ monomer consists of two Eu^III^ ions and quadruple strands of the dianionic BTP ligand (Figure 5a; the Eu^III^–Eu^III^ distance is 7.3304(6) Å). The sodium ions act as counterions to neutralize the doubly charged complexes of [Eu^III^_2_(BTP)_4_]. Two oxygen atoms of BTP ligands connect a Na^+^ and a Eu^III^ ion together (Figure 5c). The Eu^III^–Na^+^ distance is 3.791(2) Å. A Na^+^ ion is bridged to an adjacent Na^+^ ion by two water molecules, generating a linear coordination polymer (Figure 5d; Na^+^–Na^+^ distance = 3.507(3) and Na^+^–O(OH_2_) distances = 2.338(3) and 2.409(5)). All of the Eu^III^ ions are coordinated by eight β-diketonate oxygen atoms and exhibit a square antiprism (SAP) geometry (Figure 5b). The Eu–O(β-diketonate oxygen) distances are in the range of 2.363(4)–2.421(3) Å.

### Photoluminescence and CPL Properties

**Figure 6:**
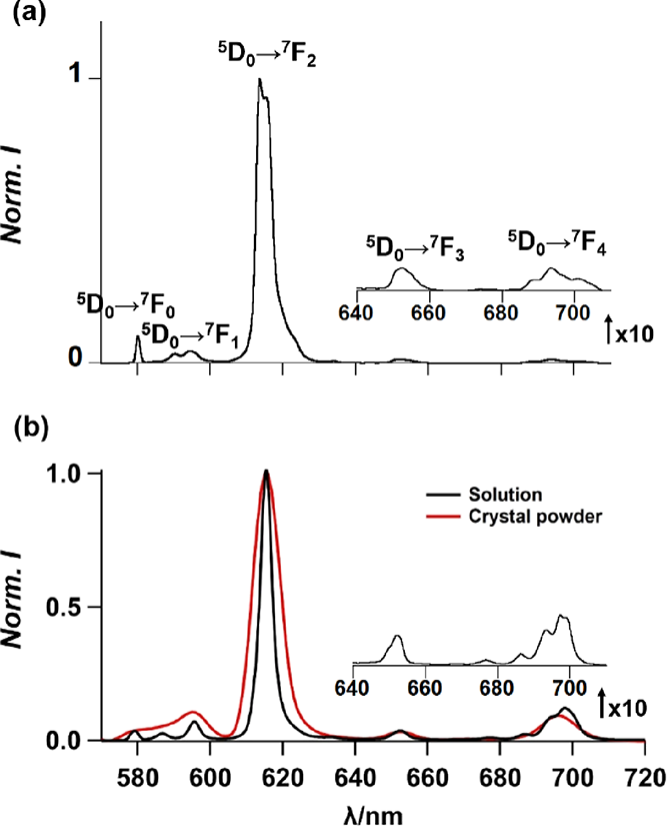
Emission spectra of (a) Eu^III^_4_(TTP)_4_(bipy)_4_(MEK)_2_(OH_2_)_2_ in chloroform (conc. = 1.7 × 10^–6^ M) and (b) [(Δ,Δ,Δ,Δ,Δ,Δ)- or (Λ,Λ,Λ,Λ,Λ,Λ)-[Eu^III^_6_(TTP)_8_(OH_2_)_6_Na_4_]_*n*_ as crystalline powder (red) and in acetone (black) at 298 K.

Upon excitation at the ligand absorption band (λ = 360 nm), Eu^III^_4_(TTP)_4_(bipy)_4_(MEK)_2_(OH_2_)_2_ and [Eu^III^_6_(TTP)_8_(OH_2_)_6_Na_4_]_*n*_ exhibit characteristics of red Eu^III^ luminescence (^5^D_0_→^7^F_J_, *J* = 0–4) in solution and in crushed crystalline powder. Their solution emission profiles are depicted in Figure 6. They display a single narrow line in the ^5^D_0_ → ^7^F_0_ emission band and several crystal-field splitting lines in ^5^D_0_ → ^7^F_1_ emission bands. Intense photoluminescence was observed in hypersensitive ^5^D_0_ → ^7^F_2_ transition, which is associated with the non-centrosymmetric nona-coordinated Eu^III^ cores.^54^ Due to the different Eu^III^ coordination environments as revealed in the crystal structures, Eu^III^_4_(TTP)_4_(bipy)_4_(MEK)_2_(OH_2_)_2_ exhibits different spectral line patterns in ^5^D_0_ → ^7^F_2_ emission bands.

**Figure 7:**
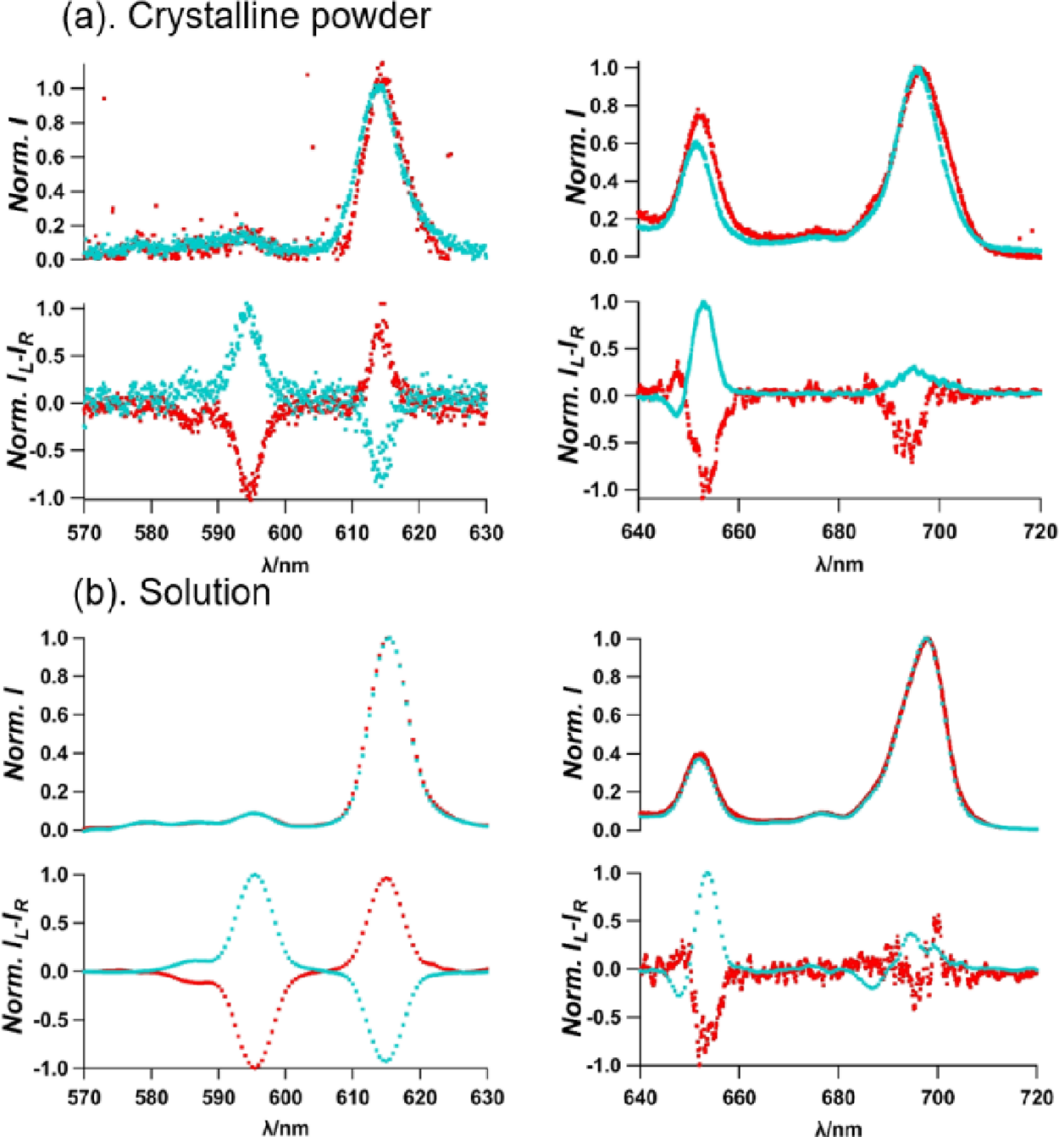
(a) [(Δ,Δ,Δ,Δ,Δ,Δ)- or (Λ,Λ,Λ,Λ,Λ,Λ)-Eu^III^_6_(TTP)_8_(OH_2_)_6_Na_4_]_*n*_ luminescence (top) and CPL spectra (bottom) recorded for crystals isolated from the conglomerate solution. The spectra of the different crystals: *C*_1_ and *C*_2_ are indicated in red and blue, respectively. (b). [(Δ,Δ,Δ,Δ,Δ,Δ)- or (Λ,Λ,Λ,Λ,Λ,Λ)-Eu^III^_6_(TTP)_8_(OH_2_)_6_Na_4_]_*n*_ luminescence and CPL spectra of the re-dissolved crystals in acetone. Solution from *C*_1_ is in red, and solution from *C*_2_ is in blue. All measurements were performed at 298 K. 570–630 nm (left); 640–720 nm (right).

Circularly polarized luminescence (CPL) occurs when an emitting species displays intrinsic chirality or stands in a chiral environment. The CPL activity is characterized by the dissymmetry factor (*g*_lum_), defined as *g*_lum_ = 2 (*I*_L_ – *I*_R_)/(*I*_L_ + *I*_R_), where *I*_L_ and *I*_R_ refer to the left and right circularly polarized intensity, respectively. We used the lab-designed CPL system with excitation illumination and emission correction at same sides of samples. Automatic correction of linearly polarized component minimizes the artifact for reliable CPL profiles of powder samples. In the solid-state CPL study, two pieces of crystals were randomly selected among samples in a vessel containing mainly homo-chiral crystals, *C*_1_ and *C*_2_, which were characterized as [(Δ,Δ,Δ,Δ,Δ,Δ)- and (Λ,Λ,Λ,Λ,Λ,Λ)-Eu^III^_6_(TTP)_8_(OH_2_)_6_Na_4_]_*n*_, respectively, after the X-ray crystallographic analysis. As the crystals gradually undergo degradation under air, CPL analysis was performed with the powder sample deposited on quartz plates. As shown in Figure 7, almost complete mirror CPL images were obtained for *C*_1_ and *C*_2_, respectively. Clear CPL profiles are shown for the specific Eu^III^ transitions, namely, ^5^D_0_ → ^7^F_1_ at approximately 596 nm and ^5^D_0_ → ^7^F_4_ at approximately 614 nm. The highest *g*_lum_ values, that is, −0.29 for *C*_1_ and +0.1 for *C*_2_, were evaluated at the magnetic dipole transition (λ = 595 nm). This is no surprise as this transition satisfies the magnetic-dipole selection rule, Δ*J* = 0, ±1 (except 0 ↔ 0), and often shows particularly large circular polarization.^40,55,56^ Several justifications can explain the difference in maximum *g* values evaluated between the two samples: it may be attributed to the different levels of degradation of each crystal after being extracted from the crystallization solution, or alternatively, it may be due to the selection of twinned crystals or crystals that do not correspond to a perfect spontaneous resolution (crystals that are intermediate between conglomerate and racemate, containing, e.g., 90% a given enantiomer). These last explanations are also consistent with the reported values of the Flack parameter, which are considerably higher than 0. In order to assess the chiral structure in the solution phase, the powders were dissolved in acetone and CPL was monitored as shown in Figure 7b. Interestingly, the initial solution phase sample showed CPL profiles that are almost identical to those in the crystalline phase with a similar intensity ratio and opposite phases at ^5^D_0_ → ^7^F_1_ and ^5^D_0_ → ^7^F_4_ transitions. Although we are not fully confident with the reliability, the magnetic dipole transition of ^5^D_0_ → ^7^F_1_ indicated *g*_lum_ values of −0.15 and +0.68 for *C*_1_ and *C*_2_, respectively.

**Figure 8:**
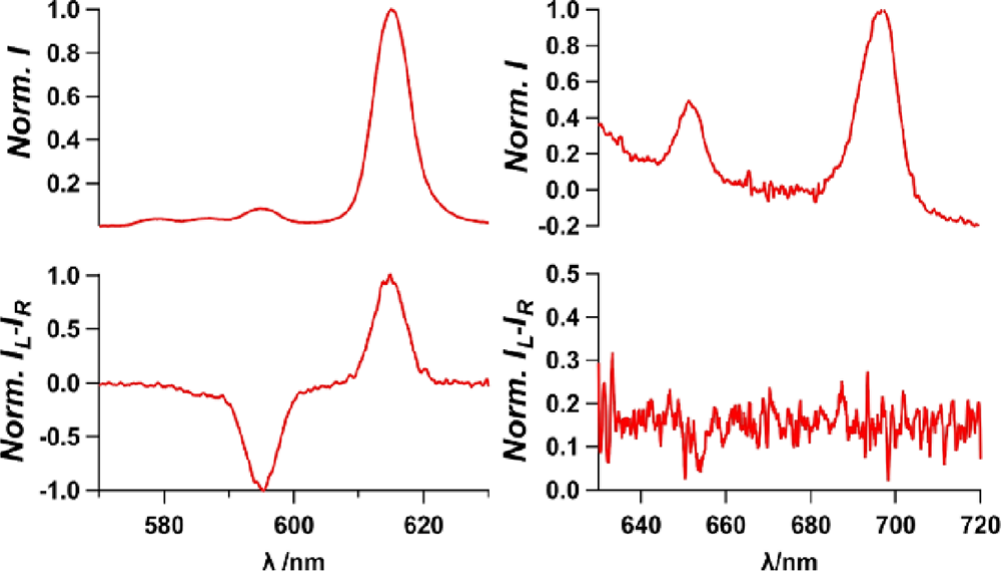
Emission spectra (top) and CPL intensity (bottom) of *C*_1_ measured after one week in acetone. 570–630 nm area (left); 640–720 nm (right).

The CPL capability of the acetone solution of *C*_1_ was further analyzed after one week, demonstrating some retention of notable CPL activity (Figure 8). The stored solution showed an identical emission profile (Figure 8) with the freshly prepared solution (Figure 7b). The CPL signals at ^5^D_0_ → ^7^F_1_ and ^5^D_0_ → ^7^F_2_ transitions were also essentially same as of the original state. The absolute value of the dissymmetry factor, *g*_lum_, at the ^5^D_0_ → ^7^F_1_ changes from −0.15 to −0.1, whereas ^5^D_0_ → ^7^F3 and ^5^D_0_ → ^7^F_4_ transitions disappear totally. It is hypothesized that the [(Δ,Δ,Δ,Δ,Δ,Δ)-/(Λ,Λ,Λ,Λ,Λ,Λ)-Eu^III^_6_(TTP)_8_(OH_2_)_6_Na_4_]_*n*_ goes dissolved with a minor modification of the coordination structure, preserving the CPL activity. The slow rearrangement/decomposition or randomization of the self-isolated chiral structure over time could be responsible for the slow decay on CPL activity (Figures 7b and 8). The multi-nuclear Eu^III^ chiral cage seems to be kinetically stable and considerably suppresses the racemization in the solution phase.

## Conclusions

In conclusion, we have successfully prepared [(Δ,Δ,Δ,Δ,Δ,Δ)- or/and (Λ,Λ,Λ,Λ,Λ,Λ)-Eu^III^_6_(TTP)_8_(OH_2_)_6_Na_4_]_*n*_ conglomerate and racemate crystals, (Δ,Δ,Δ,Δ)-/(Λ,Λ,Λ,Λ)-Eu^III^_4_(TTP)_4_(bipy)_4_(MEK)_2_(OH_2_)_2_ racemate crystals, and a [Eu^III^_2_(BTP)_4_(OH_2_)_2_Na_2_]_*n*_ achiral crystal. The conglomerate crystallization process of [Eu^III^_6_(TTP)_8_(OH_2_)_6_Na_4_]_*n*_ was associated with CF–Na^+^ interactions between the trifluoromethyl unit of the TTP ligand and Na^+^ ions. Conglomerate chiral crystals have been isolated, and their luminescence properties were investigated in the solution and solid state, revealing CPL activity with high *g*_lum_ values at the transition ^5^D_0_ → ^7^F_1_. The chiral structure was sustained even after one week in the solution phase with minor racemization.

## Experimental Section

### Synthesis and Characterization

Chemicals were purchased from Wako Pure Chemical Industries, Ltd., and used as received without further purification. ^1^H NMR and ^19^F NMR spectra were measured with JEOL ECA (600 MHz). Mass spectra were measured with mass spectrometers (JEOL AccuTOF JMS-T100LC for ESI and JMS-700 MStation for EI).

#### Synthesis of 1,3,5-Tris(3-trifluoromethyl-3-oxopropanoyl)benzene (H_3_TTP)

The two-necked round-bottomed flask was dried, evacuated, and filled up with argon gas before use. To the flask, dried THF (25 mL) was introduced. Sodium methoxide (2 mL, 0.005 M) in methanol, ethyl trifluoroacetate (1.7 mL, 14.00 mmol), and 1,3,5-triacetylbenzene (0.554 g, 2.71 mmol) were added subsequently. The reaction mixture was then stirred for 24 h. After that, the resultant mixture was poured into ice-cold water (100 mL) and acidified by hydrochloric acid (1 M) to pH of 2–3. The solution was then extracted with ethyl acetate (25 mL, three times), washed with water and brine solution, and dried with anhydrous magnesium sulfate (MgSO_4_). The precipitate was obtained from the evaporation of the resultant solution under reduced pressure. Recrystallization from isopropanol gave suitable crystals for X-ray analysis. Yield: 54.7%. EI-MS (+): *m*/*z* = 492.0263 [M^+^]. ^1^H NMR (CDCl_3_, 600 MHz, 298 K): δ 8.68 (s, 3H), 6.73 (s, 3H). ^19^F NMR (CDCl_3_, 600 MHz, 298 K): δ −76.28 (s, 9F).

#### Synthesis of [Eu^III^_6_(TTP)_8_(OH_2_)_6_Na_4_]_*n*_

A solution of NaOH (12 equiv) in methanol (5 cm^3^) was added into the solution of H_3_TTP (4 equiv) in methanol (5 cm^3^). Then, a solution of EuCl_3_·6H_2_O (3 equiv) in methanol (5 cm^3^) was added dropwise. The reaction mixture was stirred overnight. Powder was obtained after removing the solvent by a rotatory evaporator. The obtained powder was filtered, washed with water, and dried under vacuum. Recrystallization from the solvent pair of acetone and diethyl ether gave homoconfigurational conglomerate crystals of [(Λ,Λ,Λ,Λ,Λ,Λ)- or (Δ,Δ,Δ,Δ,Δ,Δ)-Eu^III^_6_(TTP)_8_(OH_2_)_6_Na_4_]_*n*_. Yield: 43.2% ^1^H NMR (CD_3_COCD_3_, 600 MHz, 298 K): δ 9.98–10.87 (m, br, 24H), −2.14–1.22 (m, br, 24H).^19^F NMR (CD_3_COCD_3_, 565 MHz, 298 K): δ −84.96 (br, 48F), −85.15(br, 12F), −85.23(br, 12F).

#### Synthesis of Eu^III^_4_(TTP)_4_(sol)_*n*_ (sol = OH_2_ or CH_3_OH)

A solution of NaOH (3 equiv) in methanol (5 cm^3^) was added into the solution of H_3_TTP (1 equiv) in methanol (5 cm^3^). Then, a solution of EuCl_3_·6H_2_O (1 equiv) in methanol (5 cm^3^) was added dropwise. The reaction mixture was stirred overnight. Water was then added to induce precipitation. The precipitate formed was filtered, washed with water, and dried under vacuum. ESI-MS(+): *m*/*z* = 2780.729 [Eu_4_(TTP)_4_(CH_3_OH)_5_(H_2_O) + Na]^+^, *m*/*z* = 2746.787 [Eu_4_(TTP)_4_(CH_3_OH)_5_ + Na]^+^. ^1^H NMR (CD_3_OD, 600 MHz, 298 K): δ 13.63 (s, 12H), 7.87 (d, *J* = 239.4 Hz, 12H). ^1^H NMR ((CD_3_)_2_CO, 600 MHz, 298 K): δ 13.87 (s, 12H), 7.95 (s, 12H). ^19^F NMR ((CD_3_)_2_CO, 565 MHz, 298 K): δ −81.49 (s, 36F). ^19^F NMR (CD_3_OD, 565 MHz, 298 K): δ −81.95 (d, *J* = 190.7 Hz, 36F). Recrystallization from dimethoxyethane (DME) and hexane gave poor-quality crystals of a racemic mixtures of (Δ,Δ,Δ,Δ)- and (Λ,Λ,Λ,Λ)-[Eu_4_(TTP)_4_(DME)_4_].

#### Synthesis of [Eu^III^_4_(TTP)_4_(bipy)_4_(MEK)_2_(OH_2_)_2_

Under the reflux condition, Eu^III^_4_(TTP)_4_(sol)_*n*_ (0.050 g, 0.018 mmol) in methanol (10 mL) was dissolved in a two-necked flask. To this solution, 2,2′-bipyridine, bipy (0.012 g, 0.077 mmol) in methanol (5 mL) was added dropwise. The reaction mixture was stirred overnight at 65 °C. Powder was obtained after removing the solvent by a rotatory evaporator. Crystallization from the solvent pair of methyl ethyl ketone (MEK) and hexane gave racemic crystals of (Δ,Δ,Δ,Δ)- and (Λ,Λ,Λ,Λ)-Eu^III^_4_(TTP)_4_(bipy)_4_(MEK)_2_(OH_2_)_2_. Yield: 77%. ESI-MS(+): *m*/*z* = 3230.347 [Eu_4_(TTP)_4_(bipy)_4_(OH_2_) + Na]^+^, *m*/*z* = 3212.051 [Eu_4_(TTP)_4_(bipy)_4_ + Na]^+^. ^1^H NMR (CD_3_COCD_3_, 600 MHz, 298 K): δ 12.77 (br, 12H), 8.28–7.82 (br, 32H), 6.8 (br, 12H). ^19^F NMR (CD_3_COCD_3_, 565 MHz, 298 K): δ −80.89 (br, 36F).

#### Synthesis of [Eu^III^_2_(BTP)_4_(OH_2_)_2_Na_2_]_*n*_

A solution of NaOH (4 equiv) in methanol (5 cm^3^) was added into the solution of H_2_BTP (2 equiv) in methanol (5 cm^3^). Then, a solution of EuCl_3_·6H_2_O (1 equiv) in methanol (5 cm^3^) was added dropwise. The reaction mixture was stirred overnight. Powder was obtained after removing the solvent by a rotatory evaporator. The precipitate formed was filtered, washed with water, and dried under vacuum. Single crystals were obtained by slow diffusion of chloroform into a solution of the sample in acetone. Yield: 35%.

### Crystallography

Conglomerate and racemic crystals of [(Δ,Δ,Δ,Δ,Δ,Δ)- or/and (Λ,Λ,Λ,Λ,Λ,Λ)-Eu^III^_6_(TTP)_8_(Na)_4_(OH_2_)_6_]_*n*_ were obtained by slow diffusion of diethyl ether into a solution of the sample in acetone. Racemic crystals of (Δ,Δ,Δ,Δ)-/(Λ,Λ,Λ,Λ)-Eu^III^_4_(TTP)_4_(bipy)_4_(MEK)_2_(OH_2_)_2_ were obtained by slow diffusion of hexane into a solution of the sample in methyl ethyl ketone. Achiral crystals of [Eu^III^_2_(BTP)_4_(Na)_2_(OH_2_)_2_]_*n*_ were obtained by slow diffusion of chloroform into a solution of the sample in acetone. A single crystal was mounted with epoxy resin on a glass fiber. The X-ray diffraction intensity was collected with a Rigaku RAXIS RAPID (3 kW) imaging plate area detector with graphite monochromated Mo Kα radiation at 123.15 K. Calculation of (Δ,Δ,Δ,Δ)-/(Λ,Λ,Λ,Λ)-Eu^III^_4_(TTP)_4_(bipy)_4_(MEK)_2_(OH_2_)_2_ was performed with the Rigaku Crystal Structure 3.8.1 software and solved by direct methods (SHELXT Version 2018/2) and expanded using Fourier techniques. The rest were solved with the SHELXT structure solution program using Intrinsic Phasing and refined with the SHELXT refinement package using Least Squares minimization. Crystal structures of (Δ,Δ,Δ,Δ)-/(Λ,Λ,Λ,Λ)-Eu^III^_4_(TTP)_4_(bipy)_4_(MEK)_2_(OH_2_)_2_ and [(Δ,Δ,Δ,Δ,Δ,Δ)- or (Λ,Λ,Λ,Λ,Λ,Λ)-Eu^III^_6_(TTP)_8_(Na)_4_(OH_2_)_6_]_*n*_ have significant A alerts due to the disordering on the trifluoromethyl units (CF_3_) of the TTP ligand and possibly on some solvent molecules.

### CPL Measurements

The CPL spectra were measured using the lab-designed CPL system consisting of an excitation laser at 375 nm, Hinds PEM-90 photoelastic modulator with a frequency of 50KHz, Hamamatsu H7732 photomultiplier tube with a signal amplifier, polarizing prism, and Shimadzu monochromator (10 cm, single grating). The system was previously calibrated, and detailed information has been discussed in a previous manuscript (*J. Am. Chem. Soc.,* 2011, **133**, 9892, *Chem. Commun*., 2012, **48**, 6025, *Synth. Met.*, 2010, **159**, 952). To realize the CPL measurements of the chiral species of Eu^III^_6_(TTP)_8_(Na)_4_(OH_2_)_6_]_*n*_, crystals were grown and randomly selected, and it took nine trials to obtain two crystals displaying opposite CPL signals.

## Supporting Information Available

Experimental details including the crystallographic parameters and refinement details, crystal structures, summary of ligand-to-ligand interactions, NMR spectra, and supporting figures for the experimental section (PDF)

## Abbreviations

CCR2: CC chemokine receptor 2
CCL2: CC chemokine ligand 2
CCR5: CC chemokine receptor 5
TLC: thin-layer chromatography
