# Supplementary material for: Conglomerate, Racemate, and Achiral Crystals of Polymetallic Europium(III) Compounds of Bis- or Tris-β-diketonate Ligands and Circularly Polarized Luminescence Study
Source: ACS Omega. 2023 Jan 31;8(6):5722–30. doi: 10.1021/acsomega.2c07310 (PMC9933189; doi:10.1021/acsomega.2c07310)
Supplement: Supplementary file 1 — ao2c07310_si_001.pdf [file ao2c07310_si_001.pdf]

Supporting Information

Conglomerate, Racemate and Achiral Crystals of Polymetallic Europium(III) Compounds of Bis- or Tris-β-diketonates Ligands and Circularly Polarized Luminescence Study

Marine Louis[a]†, Yan Bing Tan[a]†, Pablo Reine[a], Shohei Katao[a], Yoshiko Nishikawa[a], Fumio Asanoma[a], and Tsuyoshi Kawai[a]**

Graduate School of Science and Technology, Division of Materials Science, Nara Institute of Science and Technology, NAIST, 8916-5 Takayama, Ikoma, Nara 630-0192, Japan.

Table of Contents

**Table S1.** Distances of Na-Eu^{III}, Na-O, and Na-FC in [(Δ,Δ,Δ,Δ,Δ,Δ)-[$Eu^{III}_6(TTP)_8(OH_2)_6Na_4$]	**S3**
**Table S2.** Crystallographic parameters and refinement details for racemic crystal structures of $[Eu^{III}_6(TTP)_8(OH_2)_6Na_4]_n$	**S4**
**Figure S1:** Schematic representation of the structure of $Eu^{III}_6(TTP)_8(OH_2)_6Na_4]_n$ cluster	**S5**
**Figure S2.** Poor resolved racemic crystal structures of $[Eu^{III}_6(TTP)_8(OH_2)_6Na_4]_n$	**S6**
**Table S3.** Crystallographic parameters and refinement details for $H_3(TTP)$	**S7**
**Figure S3.** Poor resolved racemate crystal structure of $Eu_4(TTP)_4(DME)_4(sol)_n$	**S8**
**Table S4.** Crystallographic parameters and refinement details for (Λ,Λ,Λ,Λ)-/(Δ,Δ,Δ,Δ)-$Eu^{III}_4(TTP)_4(bipy)_4(MEK)_2(OH_2)_2$ (CCDC 2017849), and $[Eu^{III}_2(BTP)_4(Na)_2(OH_2)_2]_n$ (CCDC 2039466).	**S9**
**Figure S4-S5.** NMR Spectra	**S10-11**
**Figure S6.** Observed interactions in $Eu^{III}_4(TTP)_4(bipy)_4(MEK)_2(OH_2)$ crystal structure	**S12**

Table S1. Distances of Na-Eu^{III}, Na-O, and Na-FC in [(Δ,Δ,Δ,Δ,Δ,Δ)- [Eu^{III}_6(TTP)$_8$(OH$_2$)$_6$Na$_4$]

**Na-Eu^{III}**	**distance (Å)**	**Na-FC**	**distance (Å)**
Na_1-Eu_1	3.544(4)	Na_1-F(19)	2.709(8)
Na_1-Eu_5	3.529(4)	Na_1-F(54)	2.700(8)
Na_2-Eu_2	3.532	Na_2-F(14)	2.733
Na_3-Eu_4	3.568(4)	Na_4-F(72)	2.727
Na_4-Eu_6	3.554		
Na_5-Eu_3	3.405(6)		
**Na-O(OH$_2$)**	**distance (Å)**	**Na-O(TTP)**	**distance (Å)**
Na_1-O(1)	2.381(8)	Na_1-O(15)	2.343(8)
Na_1-O(2)	2.407(9)	Na_1-O(21)	2.498(9)
Na_2-O(27)	2.357	Na_1-O(33)	2.512(9)
Na_3-O(48)	2.47(4)	Na_1-O(39)	2.353(9)
Na_3-O(49)	2.398(9)	Na_2-O(6)	2.499
Na_4-O(56)	2.429	Na_2-O(12)	2.343
		Na_3-O(37)	2.430(8)
		Na_3-O(50)	2.400(8)
		Na_4-O(46)	2.497
		Na_4-O(55)	2.360
		Na_5-O(7)	2.683(9)
		Na_5-O(23)	2.68(1)
		Na_5-O(30)	2.68(1)
		Na_5-O(42)	2.745(9)

Table S2. Crystallographic parameters and refinement details for racemic $[(Eu^{III}{}_6(TTP)_8(OH_2)_6Na_4]_n$ （CCDC 2194127）

	$(\Delta,\Delta,\Delta,\Delta,\Delta,\Delta)/(\Lambda,\Lambda,\Lambda,\Lambda,\Lambda,\Lambda)$-$[(Eu^{III}{}_6(TTP)_8(OH_2)_6Na_4]_n$
formula sum	$[C_{144}H_{60}Eu_6F_{72}Na_4O_{54}]$
formula weight	5025.64
crystal system	monoclinic
space group	2/m
a (Å)	20.1140 (4)
b (Å)	34.7189 (6)
c (Å)	38.0705 (7)
α (deg)	90.000
β (deg)	102.036
γ (deg)	90.000
V (Å^3)	26001.5 (11)
T (K)	103.15
Z	2
ρ calcd (g cm^{-3})	1.553
R1 [I > 2σ(I)]	0.2449
wR2 [I > 2σ(I)]	N/A
Flack parameter	-

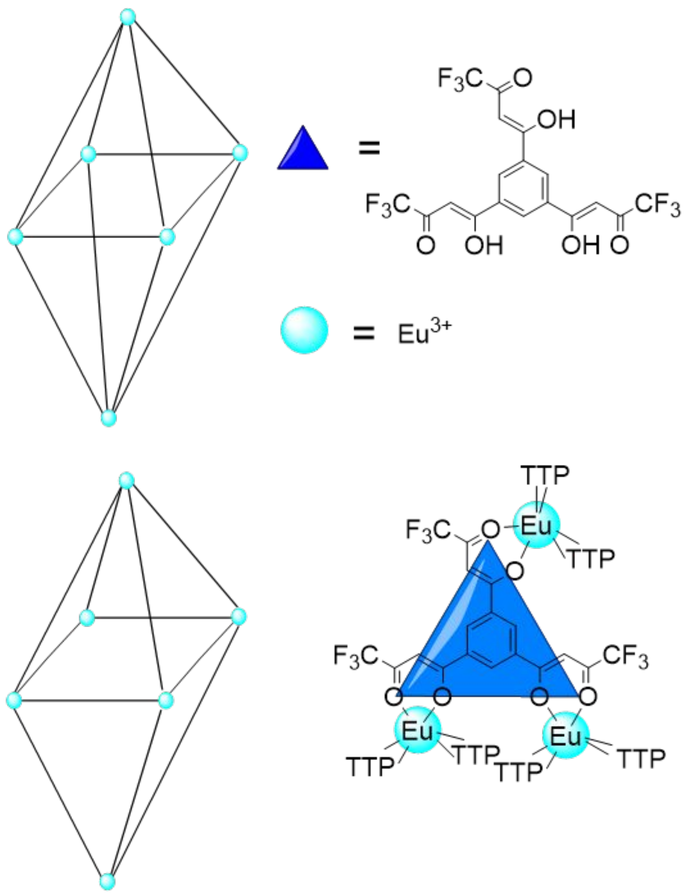

Figure S1**:** Schematic representation of the structure of $Eu^{III}{}_6(TTP)_8(OH_2)_6Na_4]_n$ cluster.

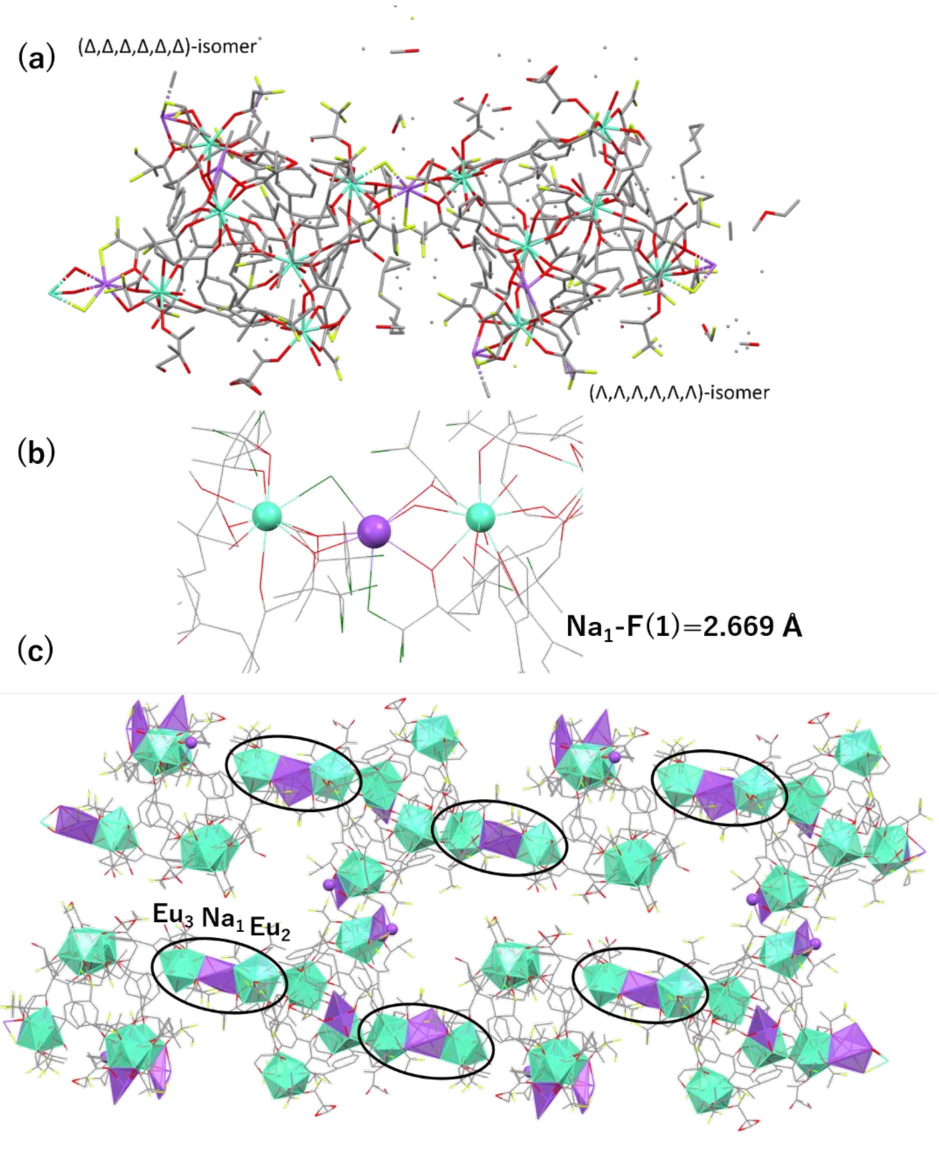

Figure S2. **(a)** Partially resolved racemic crystal structures of (Δ,Δ,Δ,Δ,Δ,Δ)- and (Λ,Λ,Λ,Λ,Λ,Λ)-$[Eu^{III}_6(TTP)_8(OH_2)_6Na_4]_n$. **(b)** A Eu-Na-Eu connection in racemic $[Eu^{III}_6(TTP)_8(OH_2)_6Na_4]_n$. (c) Crystallographic packing of the $[Eu^{III}_6(TTP)_8]$. 1-Dimensional polymer, with the connection encircled in black. Solvents and statistical error have been omitted for clarity. Light green and purple polyhedrons indicate Eu^{III} and Na^+ ions, respectively.

Table S3. Crystallographic parameters and refinement details for H_3(TTP) (CCDC 2039465).

	H_3(TTP)
formula sum	$C_{18}H_9F_9O_6$
formula weight	492.25
crystal system	monoclinic
space group	C2/c
a (Å)	85.081(4)
b (Å)	4.7869(2)
c (Å)	28.4363(13)
α (deg)	90.000
β (deg)	98.912(7)
γ (deg)	90.000
V (Å^3)	11436.6(9)
T (K)	123.15
Z	24
ρ calcd (g cm^{-3})	1.715
R1 [I > 2σ(I)]	0.0902
wR2 [I > 2σ(I)]	0.2290
Flack parameter	-

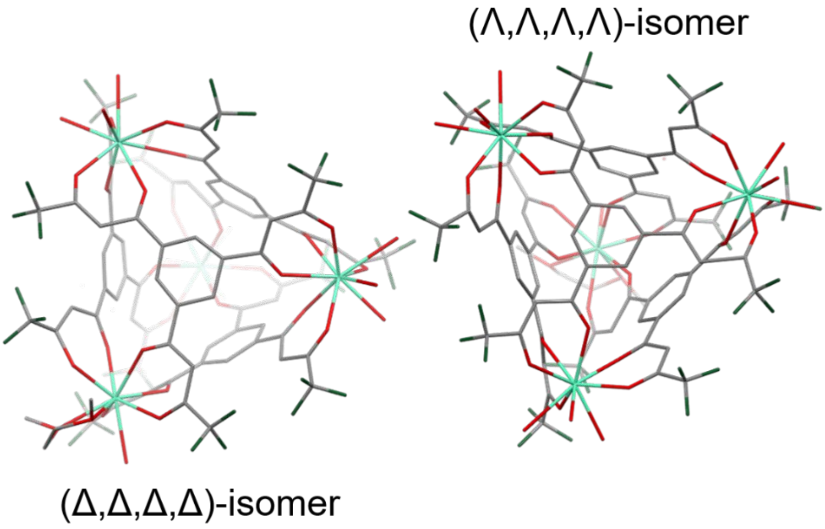

Figure S3. Poor resolved crystal structures of (Δ,Δ,Δ,Δ)- and (Λ,Λ,Λ,Λ)-$Eu_4(TTP)_4(DME)_4(sol)_n$.

Table S4. Crystallographic parameters and refinement details for (Λ,Λ,Λ,Λ)-/(Δ,Δ,Δ,Δ)-$Eu^{III}_4(TTP)_4(bipy)_4(MEK)_2(OH_2)_2$ (CCDC 2017849), and $[Eu^{III}_2(BTP)_4(Na)_2(OH_2)_2]_n$ (CCDC 2039466).

	(Λ,Λ,Λ,Λ)-/(Δ,Δ,Δ,Δ)-$Eu^{III}_4(TTP)_4(bipy)_4(MEK)_2(OH_2)_2$	$[Eu^{III}_2(BTP)_4(Na)_2(OH_2)_2]_n$
formula sum	$C_{120}H_{76}Eu_4F_{36}N_8O_{28}$	$[C_{56}H_{28}Eu_2F_{24}Na_2O_{18}]$
formula weight	3369.75	1794.69
crystal system	tetragonal	triclinic
space group	P-4c2	P-1
a (Å)	27.0056(5)	10.7742(3)
b (Å)	27.0056(5)	11.8171(3)
c (Å)	20.3638(4)	15.4795(4)
α (deg)	90	73.679(5)
β (deg)	90	87.993(6)
γ (deg)	90	69.893(5)
V (Å^3)	14851.3(5)	1772.00(11)
T (K)	123	123.15
Z	4	2
ρ calcd (g cm^{-3})	1.636	1.712
R1 [I > 2σ(I)]	0.0256	0.0393
wR2 [I > 2σ(I)]	0.0603	0.1103
Flack parameter	0.008(2)	-

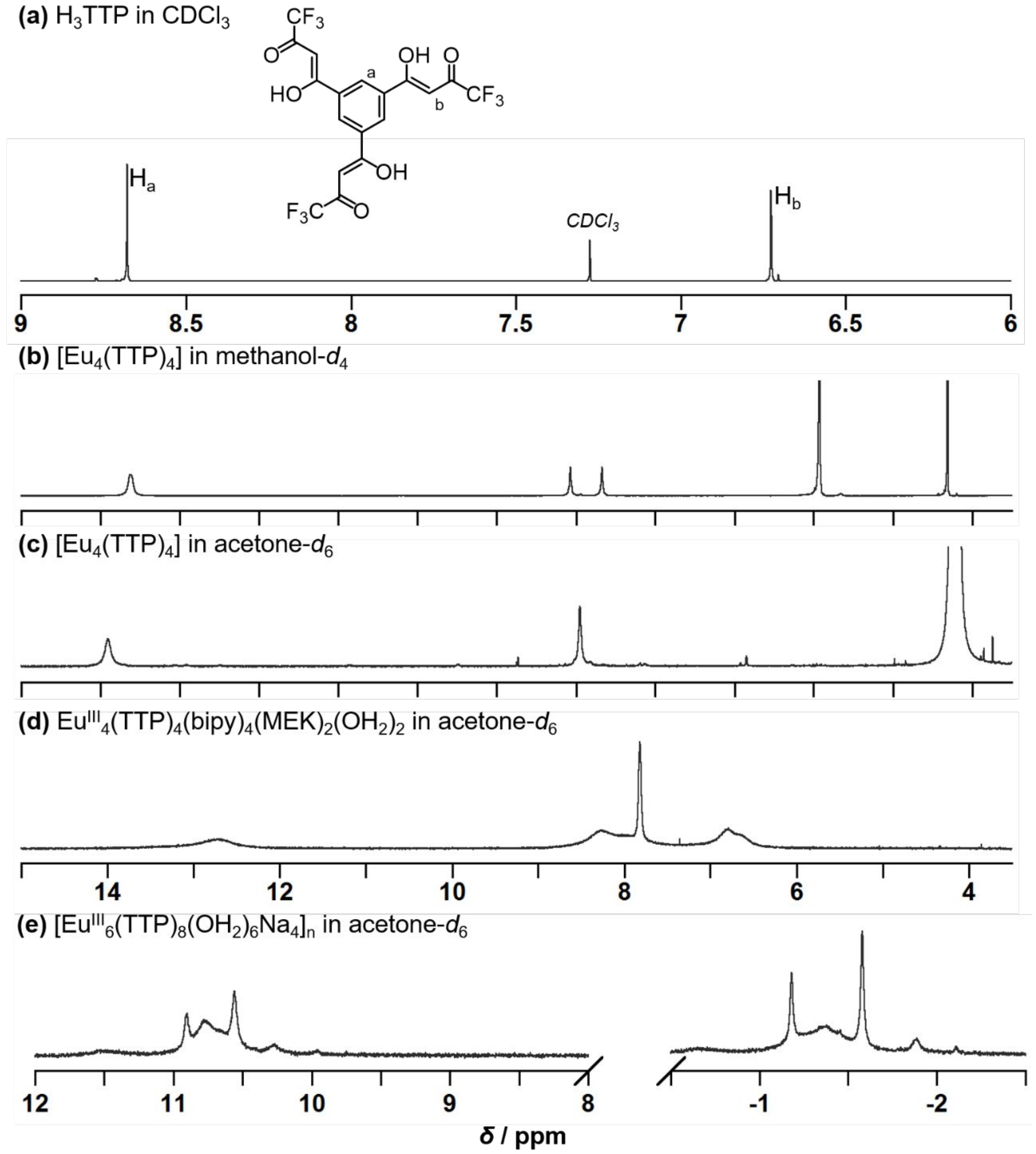

Figure S4. ^{1}H NMR spectra of (a) H_3(TTP) in $CDCl_3$, (b) [Eu^{III}_4(TTP)$_4$] in methanol-d_4, (c) [Eu^{III}_4(TTP)$_4$] in acetone-d_6, (d) Eu^{III}_4(TTP)$_4$(bipy)$_4$(MEK)$_2$(OH_2)$_2$ in acetone-d_6, and (e) [Eu^{III}_6(TTP)$_8$(OH_2)$_6$$Na_4$]$_n$ in acetone-d_6 at 298K.

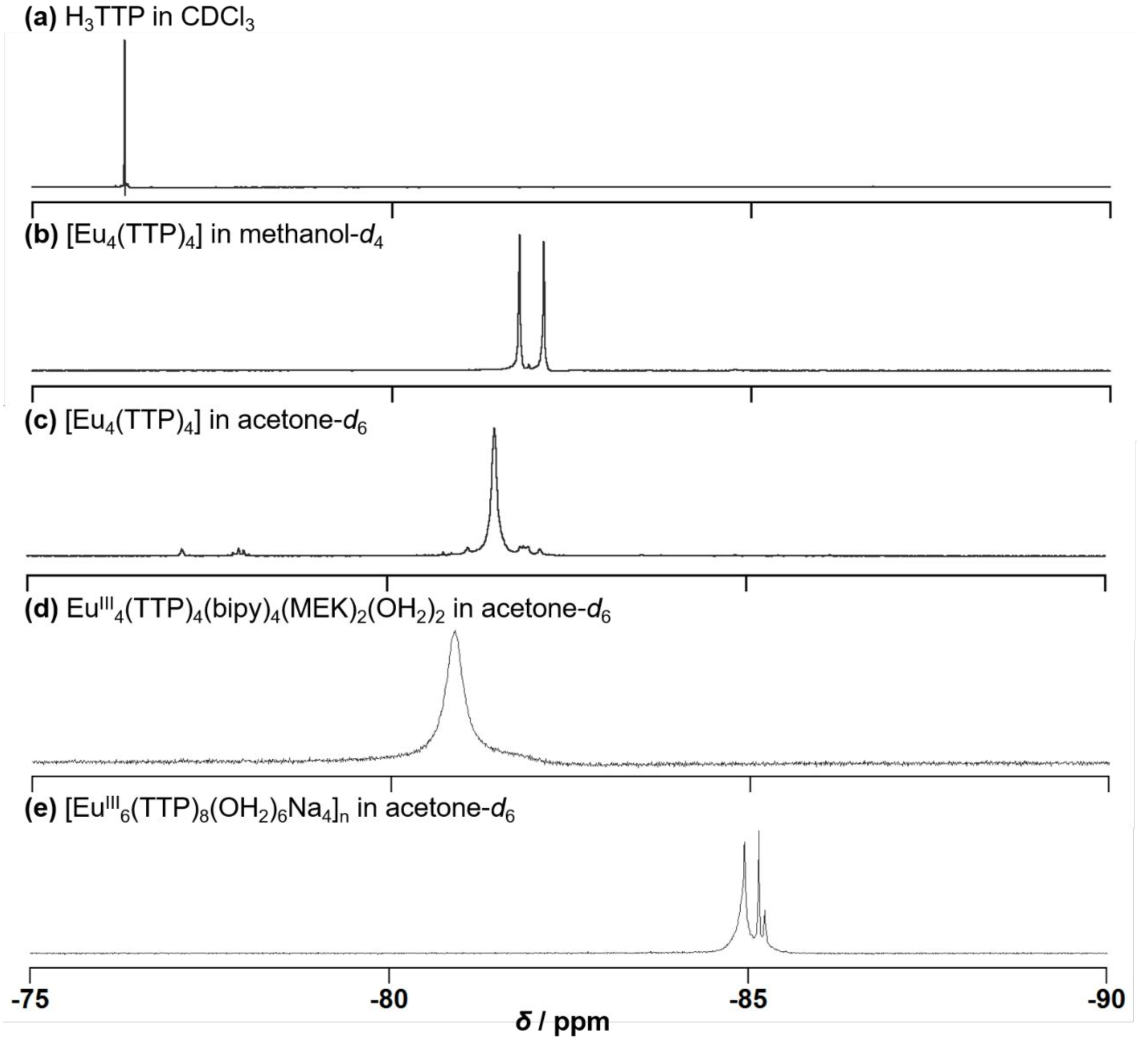

Figure S5. ^{19}F NMR spectra of (a) $H_3(TTP)$ in $CDCl_3$, (b) $[Eu^{III}_4(TTP)_4]$ in methanol-d_4, (c) $[Eu^{III}_4(TTP)_4]$ in acetone-$d_{6,}$ (d) $Eu^{III}_4(TTP)_4(bipy)_4(MEK)_2(OH_2)_2$ in acetone-$d_{6,}$ and (e) $[Eu^{III}_6(TTP)_8(OH_2)_6Na_4]_n$ in acetone-d_6 at 298K.

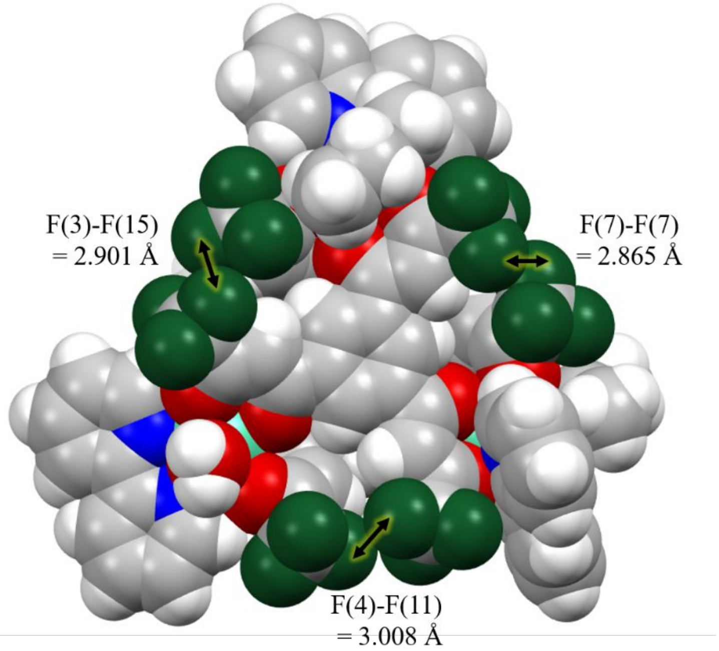

Figure S6. CF-F interactions observed in (Λ,Λ,Λ,Λ)-$Eu^{III}_4(TTP)_4(bipy)_4(MEK)_2(OH_2)_2$ crystal structure.
